# Role of Nitric Oxide in Postharvest Senescence of Fruits

**DOI:** 10.3390/ijms231710046

**Published:** 2022-09-02

**Authors:** Changxia Li, Wenjin Yu, Weibiao Liao

**Affiliations:** 1 College of Agriculture, Guangxi University, Nanning 530004, China; 2 College of Horticulture, Gansu Agricultural University, Lanzhou 730070, China

**Keywords:** nitric oxide, postharvest senescence, regulator pathway, crosstalk, genes

## Abstract

Nitric oxide (NO) acts as a gaseous signalling molecule and is considered to be a key regulator in the postharvest storage of fruits. Postharvest senescence is one of the most serious threats affecting the usage and economic value of fruits. Most recent studies have found that exogenous NO application can effectively improve the quality and prolong the shelf life of fruit postharvest by inhibiting postharvest diseases and alleviating chilling injury. Understanding the roles of NO is essential to elucidating how NO activates the appropriate set of responses to postharvest senescence. Here, we concluded that exogenous NO treatment alleviated senescence in postharvest fruit and attributed this to the following factors: (1) ethylene biosynthesis, (2) the antioxidant system, (3) polyamine metabolism and γ-aminobutyric acid (GABA) shunting, (4) cell wall metabolism, (5) sugar metabolism, (6) energy metabolism, (7) the CRT/DRE-binding factor (CBF) pathway and (8) *S*-nitrosylation. Moreover, crosstalk between NO and hydrogen sulfide (H_2_S), hydrogen peroxide (H_2_O_2_), oxalic acid (OA), arginine (Arg), GATA or plant hormone abscisic acid (ABA), melatonin (MT), and methyl jasmonate (MeJA), along with the regulation of key genes, were found to be very important in responses to postharvest senescence. In this study, we focus on the recent knowledge concerning the alleviative effect of NO on postharvest senescence, covering ethylene biosynthesis, the antioxidant system and related gene and protein expression.

## 1. Introduction

Fruits are widely consumed all over the world due to their good taste and their being rich in nutrients, such as vitamins, sugar, organic acids, and so on [1]. However, during postharvest storage, fruits are highly perishable and prone to browning, which is accompanied by a loss of taste and nutrients, directly affecting their acceptance by consumers and resulting in economic losses to fresh fruit producers [2,3]. Therefore, how to prolong the shelf life and maintain the quality of postharvest fruits has long been a focus of intense research.

Postharvest fruit senescence is a complex process affected by endogenous and exogenous environmental factors. It is well known that temperature is an important exogenous element that regulates the senescence of postharvest fruits, and low-temperature treatment is usually used as a positive effector in postharvest fruit preservation [4]. In addition, ethylene is a crucial endogenous factor in climacteric fruits because it can promote the process of senescence in harvested fruits. Thus, ethylene, as a negative factor, is usually inhibited by some new techniques in fruit storage to achieve the goal of prolonged postharvest senescence [5]. Moreover, it has been found that various signalling molecules play active roles in delaying senescence and maintaining the quality of postharvest fruits as positive effectors, such as nitric oxide (NO), hydrogen sulfide (H_2_S), melatonin (MT), etc. [6,7,8].

**Figure 1:**
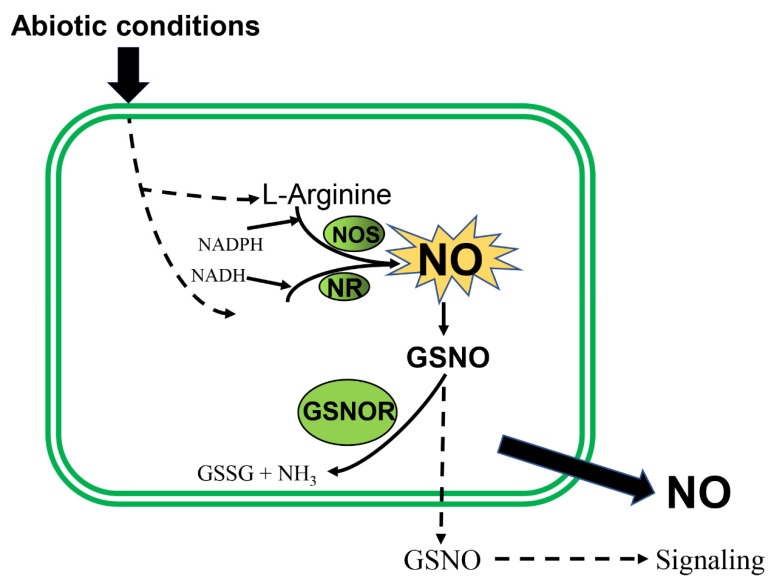
Endogenous NO production pathway in plants. NO, nitric oxide; NOS, nitric oxide synthase; NR, nitrate reductase; GSNO, S-nitrosoglutathione; GSNOR, S-nitrosoglutathione reductase; NADPH, NADPH oxidase; NADH, Nicotinamide adenine dinucleotide; GSSG, Oxidized glutathione; NH_3_, Ammonia.

Since the discovery of the biological role of NO in plants, it has been understood that it acts as a gaseous signalling molecule that participates in a series of plant physiological processes, ranging from seed germination to root development to seedling growth [9]. NO is involved in the mechanism of plant response to abiotic stress, such as drought stress, low-temperature stress, heat stress, high salinity, ozone stress and heavy metal stress [10]. In general, endogenous NO is produced mainly through oxidation and reduction pathways [11,12] (Figure 1). In the oxidation pathway, nitric oxide synthase (NOS) catalyzes the production of NO from L-arginine, and in the reduction pathway for NO production nitrites are catalyzed by nitrate reductase (NR). In addition, some abiotic conditions also stimulate the release of endogenous NO [13]. Some studies have briefly described the direct role of NO in plant senescence, including fruits and leaves, and stress induction [14,15,16].

In view of the increasing interest in studies of NO in fruits, this paper provides a critical review of the most recent work in the literature regarding various mechanisms associated with the postharvest senescence of fruits in which NO plays a role and the interaction of NO with signalling molecules. In addition, genes the expression of which is mediated by NO during the postharvest senescence process are also briefly summarized. Therefore, this review may provide new ideas for future researchers studying the regulatory role of NO in postharvest fruit senescence. Additionally, it was an objective of this study to describe in detail the regulatory mechanisms that are relevant to the postharvest preservation of fruits in which NO is involved and further accelerate research into the use of NO in delaying postharvest fruit senescence.

## 2. Effects of NO in Postharvest Fruits

### 2.1. Delaying Postharvest Senescence

Some fleshy fruits, such as peaches, tomatoes and mangoes, are well known to have relatively short storage lives due to high respiration rates and thus accelerated fruit ripening and softening, which can significantly reduce their commercial value and impede marketing and sales. However, an increasing number of studies have reported that NO could prolong the shelf life of fruit during storage, which may have a positive impact on the fruit market (Table 1). NO was found to be effective in inhibiting the surface browning of apple slices [17]. Pristijono et al. [18] investigated changes in apple slice infiltration using an NO donor diethylenetriamine-nitric oxide (DETANO) solution for 60 s and found that NO could effectively inhibit the development of surface browning and extend postharvest life. It was also reported that the softening of harvested wax apple fruit was retarded by NO [19]. During storage at 18 °C, a higher NO content could further regulate apple fruit ripening [20]. Infiltration of strawberries with DETANO solution resulted in a similar extension of shelf life to that achieved by direct fumigation with NO gas [21]. The application of NO solution S-nitrosoglutathione (GSNO) delayed the postharvest senescence of blueberries [22]. NO might enhance the resistance of tissues to decay in Chinese bayberries [23]. In kiwifruit, prolonged shelf life was regulated by NO [6]. A delay in the initiation of the senescence of kiwifruits treated with NO, which extended postharvest shelf life, was observed [24]. It was also found that sodium nitroprusside (SNP) can be used as a dipping treatment before low-temperature storage to extend the shelf life of mango fruit [25]. NO could delay the colour development, softening and ripening of mango fruits during cold storage [26]. SNP treatment could effectively decay the softening and yellowing of postharvest mango fruit [27]. Banana fruits treated with NO maintained higher firmness than the control [28]. NO application could also delay the ripening of banana slices [29]. SNP was effective in reducing pistachio browning and extending postharvest life [30]. NO could also markedly delay the occurrence of flesh browning in jujube fruits [31]. NO could be used as a fresh-keeping agent to reduce the pericarp browning of litchis before storage [32]. Cornelian cherry fruits treated with SNP displayed remarkably lower fruit browning rates during cold storage [33]. The storage life of guavas can be prolonged by postharvest immersion treatment with SNP [34]. NO has a beneficial effect on the delay of postharvest grapefruit senescence [35]. NO treatment also significantly improved the shelf life of harvested pointed gourds [36]. Fruit ripening of harvested persimmon was significantly delayed by NO [37]. Singh et al. [38] reported that NO fumigation was effective in reducing skin colour changes and retarding softening in Japanese plums. SNP treatment is an easy method for the application of NO to extend the shelf life of plums during cold storage [39]. NO treatment may delay the senescence of peach fruits under cold conditions [40]. The application of NO could be a potential method for treating harvested tomato fruits in order to delay ripening [41]. Similarly, exogenous NO treatment was found to be a potential method for extending the postharvest life of citrus fruit [42]. NO treatment could effectively delay the softening and ripening of papaya fruit [43]. NO treatment could also effectively retard the ripening of oranges during storage [44].

### 2.2. Improving Postharvest Quality

More and more research has focused on the application of exogenous NO to improve the quality of postharvest fruits in recent years. Peach fruits fumigated with NO exhibited changes in colour, soluble solid contents (SSCs), titratable acidity (TA) and SSC/TA ratios during cold storage [5]. Guava fruits under NO treatment exhibited the minimum loss of chlorophyll, SSC and acidity, and a slower increase in carotenoid pigments [34] (Table 1). NO could increase the contents of vitamin C, organic acids and total soluble solids (SSs) in grapefruits during cold storage, suggesting that NO may have potential applications in postharvest treatments to maintain fruit quality [35]. NO application effectively maintained chlorophyll and suppressed the yellow colour development undergone by pointed gourds [36]. Preharvest peach fruits treated with NO exhibited higher levels of vitamin C compared to the control [45]. NO treatment significantly retarded increase in TSS content and decrease in TA content in winter jujubes during storage [31]. NO fumigation was effective in restricting changes in fructose, glucose, sucrose, sorbitol and TA contents and in skin colour during storage at 0 °C [26,38]. NO-treated mangos maintained higher contents of SSC and TA through the delay of ripening [27]. Throughout litchi storage, NO treatment effectively delayed loss of total SS, TA and ascorbic acid (ASA) contents in fruits [32]. Retarded colour change of peel and higher TA and TSS contents of pulp were shown in the ripening process of NO-treated banana fruits [46]. SNP treatment retained higher levels of TA and ASA in postharvest apples [47]. NO could protect kiwifruits against oxidative damage by maintaining contents of vitamins C and E during cold storage [48]. Exogenous NO treatment could improve the ASA and TA contents and delay increase in total SS contents in citrus fruits during storage at 20 °C [42]. NO significantly maintained a lower total anthocyanin content and a higher total phenol content, and delayed increase in SS and decrease in vitamin C contents in Chinese winter jujubes [49]. NO had a significant impact on the soluble sugar contents of peach fruits in room-temperature storage [50]. NO increased SSC⁄TA ratios, decreased SS contents and increased the vitamin C and E contents of kiwifruits, indicating that NO is effective in maintaining fruit quality during 20 °C storage [24]. NO treatment was responsible for higher levels of TA, soluble proteins, ASA and reducing sugars and lower levels of SSC in Hami melons, and retarded their ripening during storage [44]. Higher sucrose contents in peach fruits were sustained by NO treatment under cold conditions [40]. NO applications also slowed increase in SSC contents and SSC/TA ratios and decreased loss of TA and ASA contents in oranges during cold storage [51].

### 2.3. Inhibiting Postharvest Diseases

NO treatment effectively controlled brown rot disease caused by *Monilinia fructicola* in peach fruits [52]. Zheng et al. [53] and Table 1 show that lesion sizes in tomato fruits with *Botrytis cinerea* were reduced by 93.3 and 80.9% three and four days after NO inoculation, respectively, suggesting that NO could mitigate disease symptoms during storage. Lai et al. [41] showed that expression levels of the fruit-ripening-related genes *LeACS2*, *LeACS4*, *LeACO1*, *LePG*, *LePhy1* and *LePM* were regulated by NO treatment, resulting in an increase in the resistance of tomato fruits to gray mold rot caused by *Botrytis cinerea*. Lesion development on mango fruits inoculated with *Colletotrichum gloeosporioides* was effectively suppressed by NO treatment during storage. NO treatment also significantly reduced natural anthracnose incidence in ripened mango fruits at ambient temperature [27]. SNP could markedly suppress the mycelial growth and spore germination of *Colletotrichum gloeosporioides*, resulting in delayed senescence in postharvest mango fruits [14]. NO treatment was a promising approach to improve resistance against fungal pathogens and maintain the quality of custard apples after harvest [47]. The resistance of pitaya fruit against anthracnose was also effectively enhanced by NO [54]. Use of exogenous NO was a promising method for inducing the disease resistance of fruits to fungal pathogens and extend the postharvest life of citrus fruits [42]. Liu et al. [44] indicated that NO treatment could effectively reduce disease incidence in oranges inoculated with *Collletotichum goeosporioides* and inhibit the increase in lesion diameters during storage.

### 2.4. Alleviating Chilling Injury

Ghorbani et al. [35] reported that NO could reduce chilling injury (CI) to harvested peach fruits during storage. NO reduced the incidence of CI by 1.5- to 1.7-fold in mango fruits [25] (Table 1). CI symptoms were significantly lower in NO-fumigated fruit than in non-fumigated fruit [38]. NO treatment significantly reduced the CI indexes of mango fruits during cold storage [26]. NO successfully alleviated CI in orange fruits [55]. NO treatment improved the chilling tolerance of banana fruits, as shown by reduced CI indexes after cold storage [46]. Application of NO could effectively reduce chilling injury to banana fruits [56]. NO could significantly control CI and improve resistance against chilling stress [57]. In Chinese bayberries, fumigation with NO inhibited ethylene release and disease incidence during cold storage [23]. NO treatment could alleviate and delay CI in cold-stored Hami melon fruits [58].

## 3. The Regulatory Pathways Prolonged by NO during Fruit Storage

### 3.1. Ethylene Biosynthesis

**Figure 2:**
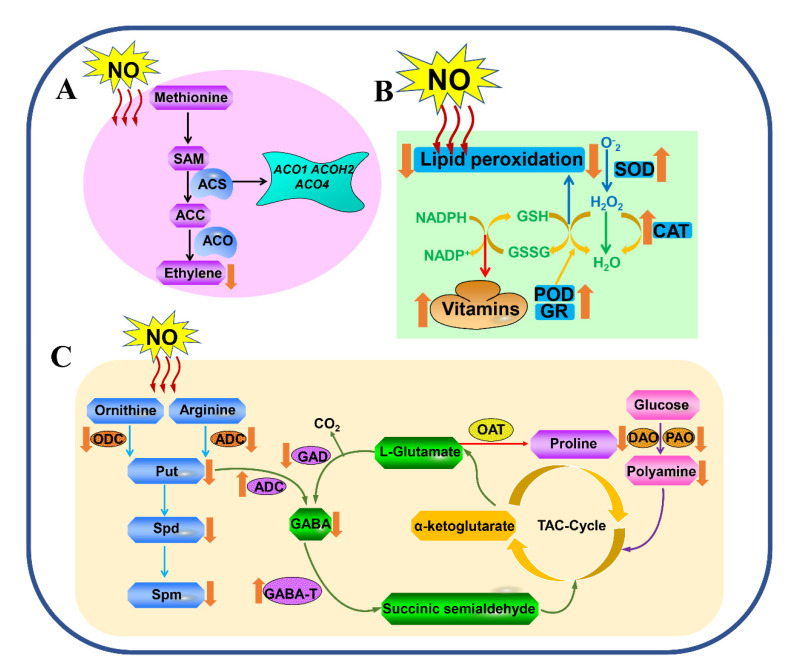
The regulatory mechanisms involving NO that are related to the postharvest senescence of plants: (**A**) ethylene biosynthesis, (**B**) the antioxidant system, (**C**) polyamine metabolism and γ-aminobutyric acid (GABA) shunting. SAM, S-adenosyl-methionine; ACS, 1-aminocyclopropane-1-carboxylate synthase; ACO, ACC oxidase; NO, nitric oxide; SOD, superoxide dismutase; CAT, catalase; POD, peroxidase; GR, Glutathione reductase; NADPH, NADPH oxidase; GSH, glutathione; GSSG, Oxidized glutathione; O^−^_2_, Superoxide radicals; H_2_O_2_, hydrogen peroxide; Put, putrescine; Spd, spermidine; Spm, spermine; ADC, arginine decarboxylase; DAO, diamine oxidase; PAO, polyamine oxidase; GABA, γ-aminobutyric acid.

It is widely known that ethylene plays a vital regulatory role in the senescence process of harvested fruits [1]. NO, as a signal molecule regulator, is thought to be closely associated with inhibition of ethylene production in events that prolong the shelf life of fruits [67] (Figure 2). In NO-treated tomato fruits, expression levels of the *LeACO1*, *LeACOH2* and *LeACO4* genes were decreased and delayed [59] (Table 1). Lai et al. [41] also showed that NO suppressed rates of ethylene production during tomato fruit storage. NO fumigation inhibited ethylene biosynthesis in mango fruits by restraining 1-aminocyclopropane-1-carboxylate synthase (ACS) and ACC oxidase (ACO) activities, leading to reduced ACC contents during cold storage [26]. Subsequently, to further verify the roles of NO in postharvest mango fruits, Barman et al. [25] found that with dipping in SNP the rate of ethylene production in mango fruits was decreased by 117–270% compared to the control fruit dipped in distilled water. In apples, SNP treatment could increase the NO contents of harvested fruits during storage, while higher NO contents could further regulate fruit senescence by regulating ethylene production during storage at 18 °C [20]. Tareen et al. [5] indicated that NO exhibited synergistic effects in the reduction of ethylene production during peach fruit cold storage. In pear storage, the expression levels of the *PcACS* and *PcACO* genes were inhibited by MT and NO treatment [60]. To sum up, the inhibition of ethylene by NO-mediated ACC/ACS activity is not directly brought about through signal regulation.

### 3.2. Antioxidant System

The softening and senescence of postharvest fruits are usually accompanied by the generation of reactive oxygen species (ROS) [68]. Antioxidants significantly combat the oxidation of a series of substrates [69] and protect different biomolecules from the effects of damaging ROS by reacting with free radicals and preventing oxidation processes. Fruits are known to be rich sources of natural antioxidants either in the form of enzymes, such as superoxide dismutase (SOD), catalase (CAT), peroxidase (POD) and polyphenol oxidase (PPO), or in the form of non-enzymes, such as vitamins, including alpha tocopherol, carotenoids and ASA [69]. NO acts as a highly reactive free radical gas that participates in the resistance of fruits to senescence. Pistachios treated with NO maintained higher SOD, PPO and POD activities, which led to delayed hull browning [30] (Figure 2). NO significantly reduced lipid peroxidation as well as malondialdehyde (MDA) and hydrogen peroxide (H_2_O_2_) contents in grapefruits during cold storage. Simultaneously, the activities of antioxidant enzymes, such as POD, APX, SOD and catalase (CAT), were also enhanced by NO during this process [35]. Jujubes treated with NO exhibited higher activities of CAT, SOD, GR and APX [31] (Table 1). Simultaneously, reductions in ASA and glutathione (GSH) contents and increases in superoxide anion (O^2−^) production and H_2_O_2_ contents were also delayed by NO treatment [31]. ROS accumulation and the lipid peroxidation of membranes were alleviated by NO through the induction of APX, SOD, POD, CAT and GR activities in the peel and pulp of table grapes [3]. NO successfully alleviated CI in orange fruits by inducing antioxidant responses, whilst maintaining fruit quality by decreasing H_2_O_2_ contents and lipid peroxidation [55]. The minimum loss of phenols, flavonoids, antioxidant capacity and radical scavenging activity were exhibited by guava fruits treated with NO [34]. Application of SNP retained greater total antioxidant activities and phenolic compound contents in harvested persimmon fruits [37]. During storage, contents of total flavonoids, phenolics, hydroxyproline-rich glycoproteins and lignin were elevated by NO in kiwifruit [6]. NO could also protect kiwifruits against oxidative damage caused by ROS during storage [48]. Contents of ASA and glutathione were improved by NO during the postharvest storage of blueberries [22]. Application of NO could effectively reduce CI in banana fruits by decreasing O^2−^ production and H_2_O_2_ contents, increas activities of POD, SOD, CAT and APX, and up-regulating expression levels of the *MaPOD, MaSOD*, *MaCAT* and *MaAPX* genes [56]. Chinese bayberry fumigation with NO delayed the decrease in total phenolic contents and DPPH radical scavenging activities, while increases in both the rate of O^2−^ production and H_2_O_2_ content were delayed, followed by increases in the activities of SOD, CAT and APX during storage [23]. Kiwifruits treated with NO had lower contents of soluble solids and MDA and higher contents of vitamins C and E during 20 °C storage [24]. Inhibition of the browning of peach slices by NO via the antioxidant system was discussed by Zhu et al. [62]. NO also effectively decreased the production of MDA, O^2−^ and H_2_O_2_ in longkong fruits during storage [57]. Meanwhile, NO-treated longkong fruit pericarps exhibited lower activities of browning-related enzymes, including PAL and PPO, and higher activities of antioxidant enzymes, including POD, SOD, CAT and GPX, than the control, suggesting that NO could significantly prolong the shelf life of longkong fruit during cold storage [57]. Application of NO effectively suppressed lignin formation and electrolyte leakage and maintained contents of chlorophyll and phenolics as well as membrane integrity in pointed gourds during storage. The activities of PAL and LOX enzymes in pointed gourds were also influenced positively by NO application during this process [36]. NO was positively correlated with pericarp browning in litchis due to reduced MDA contents [32]. Wang et al. [46] showed that activities of POD, CAT and APX were increased by NO in banana fruits after cold storage. Additionally, the antioxidant activities of DPPH and FRAP were enhanced by NO in custard apples during storage [47].

### 3.3. Polyamine Metabolism and GABA Shunting

Polyamines (PAs) mainly include spermidine (Spd), putrescine (Put) and spermine (Spm), which are widely found in plant tissues. In the biosynthesis pathway of Pas, the decarboxylation of ornithine and arginine is catabolized by ornithine decarboxylase (ODC) and arginine decarboxylase (ADC), respectively, to produce Put [70] (Figure 2). Spd and Spm can be synthesized from Put by the sequential addition of aminopropyl groups [71]. Diamine oxidase (DAO) and polyamine oxidase (PAO) catalyze the degradation of polyamines [72,73]. Polyamine metabolism is closely associated with γ-aminobutyric acid (GABA) and proline. Briefly, Put can be converted into GABA, catalyzed by DAO [72,73,74] (Figure 2). In recent years, the regulatory roles of PAs and GABA shunting in postharvest fruit senescence have been well established [61] (Table 1). Wang et al. [61] indicated that NO-induced chilling tolerance might be ascribed to the enhanced catabolism of PAs, GABA and proline in banana fruits. Cherry fruits treated with NO and GABA exhibited significantly lower fruit browning rates during cold storage, which may be ascribed to lower MDA and H_2_O_2_ contents and LOX activity and higher CAT, SOD, APX and GR activities, suggesting that applications of NO and GABA may be promising strategies for delaying the postharvest senescence of fruits [33].

### 3.4. Cell Wall Metabolism

NO mediated the activities of cell-wall-softening-related enzymes, which could effectively inhibit the softening of papaya fruits [43]. SNP is a simple NO application method for inhibiting fruit softening, maintaining fruit quality and prolonging shelf life by interfering with PAL and PME activities in ‘Santa Rosa’ plums during cold storage [39] (Table 1). NO-mediated cell wall metabolism inhibited the browning of peach slices by increasing PAL activity [62]. NO increased the activity of cell-wall-metabolism-related enzymes, such as CoA ligase (4CL), phenylalanine ammonia-lyase (PAL), polyphenol oxidase (PPO), β-1,3-glucanase (GLU) and chitinase (CHI), which might be beneficial for the retardation of senescence in pitaya fruits during storage [54]. NO also increased the activities of PAL, POD and β-1,3-glucanase during kiwifruit storage [6].

### 3.5. Sugar Metabolism

Soluble sugars, such as fructose, glucose and sucrose, are considered to be signal molecules that participate in the senescence of harvested fruits. Shi et al. [63] (Table 1) found that red raspberries treated with NO showed increased sucrose synthase (SS), sucrose phosphate synthase (SPS) and neutral invertase (NI) activities; decreased acid invertase (AI) activity; and higher fructose, glucose and sucrose contents. It has been reported that NO improved the quality of apple fruits by increasing the activities of sucrose synthase synthesis (SS-s) and SPS and decreasing sucrose synthase cleavage (SS-c), NI and AI activities, following which the expression levels of the *MdSPS*, *MdSS*, *MdNI*, *hexokinase (HK)* and *fructokinase (FK)* genes were significantly up-regulated [64]. Similarly, NO sustained higher sucrose contents and lower glucose and fructose contents in peach fruits under cold conditions by enhancing SPS activity and the expression levels of the *PpaSPS1/2* gene and decreasing SS-c activity by reducing expression levels of the *PpaAI1* sucrose-cleaving enzyme gene [40].

### 3.6. Energy Metabolism

Sugar is important not only as a factor determining fruit quality but also as a substrate for energy supply. Maintaining adequate energy supply is essential for delaying the postharvest senescence of fruits. NO could extend the duration of cold storage time for banana fruits by increasing contents of energy charge and adenosine triphosphate (ATP) and the activities of Ca^2+^-ATPase, H^+^-ATPase, succinic dehydrogenase (SDH) and cytochrome C oxidase (CCO) enzymes [46] (Table 1).

### 3.7. CBF Pathway

It is reported that CRT/DRE-binding factors (CBFs), as transcription factors, improve the chilling tolerance of fruits, while expression of the cold response gene (COR) gene is activated by recognition and binding of the C-repeat/dehydration-responsive element (CRT/DRE) cis-element [75]. Zhao et al. [65] showed that treatment of tomatoes with SNP resulted in up-regulation of the expression of stress response genes via enhanced expression of the *LeCBF1* gene, which thereby enhanced tolerance to cold stress during storage. Hami melon fruits fumigated with NO gas showed up-regulated expression levels of the *CmCBF1* and *CmCBF3* genes, which led to the alleviation of chilling injury during cold storage [58] (Table 1).

### 3.8. S-Nitrosylation

Protein *S*-nitrosylation, as a key post-translational modification, has been reported in the processes of fruit storage, salt stress and so on [66,76]. NO inhibited the gene expression, *S*-nitrosylation and activity of GSNOR, demonstrating that *S*-nitrosylation of GSNOR proteins was effective in prolonging the storage life of peach fruits and preserving them against disease; in addition, NO and GSNOR inhibitors up-regulated the relative expression of systemic acquired resistance (SAR)-related genes, including the non-expressor of PR1 (NPR1), pathogenesis-related gene 1 (PR1) and TGACG-binding factor 1 (TGA1) [66] (Table 1). Studies on the way in which NO-mediated *S*-nitrosylation modulates postharvest fruit senescence are quite limited. Therefore, it will be necessary for future studies to examine the NO-mediated *S*-nitrosylation of specific proteins and specific *S*-nitrosylation sites in this process.

## 4. Potential Networks of Crosstalk Involving NO and Other Signalling Molecules in the Regulation of Postharvest Fruit Senescence

**Figure 3:**
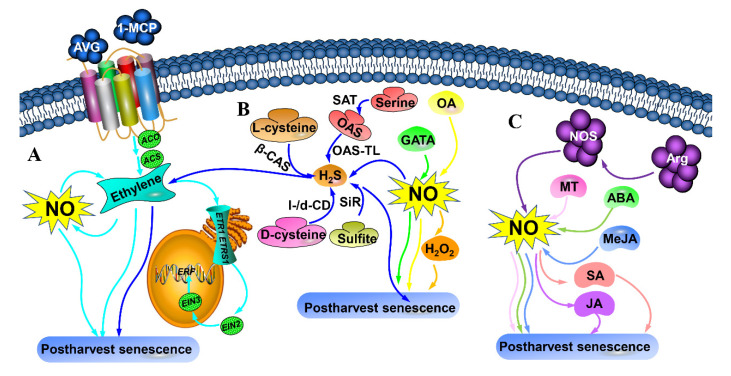
Schematic model of the interaction among H_2_, H_2_S, H_2_O_2_ and other plant hormones in postharvest senescence. (**A**) NO interacts with ethylene, (**B**) NO interacts with H_2_S, H_2_O_2_, GATA or OA, (**C**) NO interacts with plant hormones. AVG, aminoethoxyvinylglycine; 1-MCP, 1-methylcyclopropene; Arg, arginine; OA, oxalic acid; NOS, nitric oxide synthase; ABA, abscisic acid; NO, Nitric oxide; H_2_S, hydrogen sulfide; H_2_O_2_, hydrogen peroxide; MT, Melatonin; MeJA, Methyl jasmonate; GABA, γ-aminobutyric acid; l-/d-CD, 1-/d-cysteine desulfhydrase; OAS-TL, O-acetylserine (thiol)lyase; SiR, sulfite reductase; β-CAS, β-cyanoalanine synthase; JA, jasmonic acid; SA, salicylic acid.

Previous studies have shown that NO can interact with other molecules to extend the shelf lives of preharvest fruits, suggesting that NO signal transduction is not generally a linear pathway. Here, we provide a brief overview of the interactions between NO and H_2_S, H_2_O_2_, plant hormones and other signalling molecules during storage (Figure 3).

Peach fruits fumigated with NO combined with the ethylene inhibitor aminoethoxyvinylglycine (AVG) exhibited longer storage lives compared to those that received NO treatment alone [5] (Figure 3A). Wu et al. [23] showed that combined treatment with 1-methylcyclopropene (1-MCP) and NO had a functional synergistic effect in peach fruits. Ethylene-related genes, including *MiACS*, *MiACO*, *MiETR1*, *MiEIN2*, *MiERS1* and *MiERF*, might be involved in the NO and SA signal induction pathways during storage [77] (Figure 3A). Steelheart et al. [78] demonstrated that NO improved the effect of 1-MCP on the extension of the storage life of postharvest tomatoes (Figure 3A). Zhu et al. [79] also indicated that combinatorial treatment (NO+H_2_S) maintained a superior quality of peach fruits by inhibiting ethylene synthesis and cell wall metabolism (Figure 3B). NO-mediated H_2_S metabolism regulated the postharvest senescence of peach fruits by reducing endogenous H_2_S, sulfite and Cys contents; decreasing the activities of O-acetylserine (thiol)lyase (OAS-TL), l-/d-cysteine desulfhydrase (l-/d-CD) and sulfite reductase (SiR); and increasing β-cyanoalanine synthase (β-CAS) activity [80] (Figure 3B). H_2_O_2_ was required for NO-mediated disease resistance in harvested tomato fruits during storage [81] (Figure 3B). Mohamed et al. [51] reported that postharvest treatments of 1 mM SNP combined with 10 mM oxalic acid (OA) or 2% H_2_O_2_ are good potential strategies for delaying senescence as well as maintaining the quality of oranges during cold storage (Figure 3B). Arg-mediated NOS pathway activity improved the quality and delayed the postharvest senescence of strawberry fruits [82] (Figure 3C). Exogenous NO enhanced the chilling resistance of harvested peach fruits by inducing SA or JA signalling [54] (Figure 3C). MeJA may function upstream of and depend on NO in the signalling pathway that activates chilling tolerance in cucumber during storage [83] (Figure 3C). Exogenous ABA application could enhance lily bulb resistance to cold-induced oxidative stress by improving the efficiency of enzymatic and non-enzymatic systems which were found to be partially mediated by NO [4] (Figure 3C). MT acted as an upstream signal of NO synthesis and delayed postharvest senescence in pear fruits [60] (Figure 3C).

## 5. Modulation of Gene Expression by NO during Fruit Storage

As mentioned above, a series of potential mechanisms involving NO have been confirmed in the delayed senescence of postharvest fruits. Thus, some genes must inevitably be changed following exposure to NO (Table 2). For example, NO induced *VvSOD*, *VvCAT*, *VvPOD2* and *VvGR* expression [3]. Banana fruits treated with NO exhibited higher expression levels of *MaSOD*, *MaCAT*, *MaPOD* and *MaAPX* than the control during storage [56]. NO could delay kiwifruit senescence during storage, in which relative expression of the *POD*, *PAL* and *CHT* genes was up-regulated [6]. The expression of *SsC4H* was down-regulated in the NO-retarded cottony softening of harvested wax apple fruits [19]. NO also up-regulated relative expression of the *LeACO1*, *LeACOH2* and *LeACO4* genes during tomato storage [71]. The expression levels of *LeACS2*, *LeACS4*, *LeACO1*, *LePG*, *LePhy1* and *LePME* were regulated by NO during cold storage [41]. MT plus NO treatment inhibited the expression levels of the *PcACS* and *PcACO* genes, decreased the rate of up-regulation of *PcCel* and *PcPG*, and up-regulated the relative expression of *PcNOS*, implying that MT, as an upstream signalling molecule of NO, delayed senescence in pear fruits [60]. Additionally, NO treatment protected tomatoes from cold injury by inducing expression of the *LeCBF1* gene [65]. NO also could prolong the storage life of Hami melon fruits by up-regulating the expression levels of the *CmCBF1* and *CmCBF3* genes [58]. NO improved the quality of apple fruits by up-regulating the relative expression of the *MdSS*, *MdSPS*, *MdNI*, *FK* and *HK* genes [64]. Similarly, NO maintained the high quality of peach fruits during cold storage by up-regulating the SPS-related gene *PpaSPS1/2* and down-regulating the SS-c-related gene *PpaAI1* [40]. Moreover, NO inhibited gene expression of *GSNOR* and up-regulated expression of SAR-related genes, including *NPR1*, *PR1* and *TGA1*, suggesting that S-nitrosylation of GSNOR contributed to improved shelf life in peach fruits [66].

## 6. Conclusions and Future Perspective

In conclusion, in fruits, NO could delay postharvest senescence and maintain postharvest quality by inhibiting postharvest diseases and alleviating chilling injury. The regulation of ethylene biosynthesis, the antioxidant system, polyamine metabolism, GABA shunting, cell wall metabolism, sugar metabolism, energy metabolism, the CBF pathway and *S*-nitrosylation by NO are essential strategies for prolonging the storage life of fruits. Additionally, we have also reviewed the crosstalk mechanisms that are associated with applications of exogenous NO, H_2_S, H_2_O_2_, OA, Arg, GATA, plant hormone ABA, MT and MeJA in postharvest fruit preservation. Moreover, NO can regulate the expression of responsive genes during this process. These insights might help guide future postharvest fruit preservation strategy design, ultimately leading to effective preservatives better suited to our agricultural requirements.

Future studies regarding the regulatory role of NO in postharvest senescence should focus on the molecular details of the particular pathways that are activated in fruits. Nowadays, although it is a well-known fact that NO interacts with other gas signalling molecules or phytohormones in response to the postharvest senescence of fruits, the signal transduction pathway involved in this crosstalk mechanism still needs to be further elucidated. Clearly, *S*-nitrosylated proteins are quite limited during postharvest senescence. Future work is needed to detect more *S*-nitrosylated candidates in postharvest fruit senescence in order to understand the complicated functions of *S*-nitrosylation during this process. More research work will improve knowledge concerning possible applications of NO solutions to postharvest horticultural products with the aim of enhancing their quality and economic value.

## Figures and Tables

**Table 1:** The effects of NO on postharvest senescence in fruits.

Materials	Treatment	Storage	Effects	Mechanisms	References
Apple slices	NO fumigation (1 h, 10 μL·L^−1^)	10 °C	Delaying postharvest senescence		[17]
Apple slices	Infiltration with DETANO solution for 60 s	RT	Delaying postharvest senescence		[18]
Wax apple	NO fumigation (2 h, 10 μL·L^−1^)	4 ± 0.5 °C	Delaying postharvest senescence		[19]
Apple	SNP spray (50 μM)	18 ± 1 °C	Delaying postharvest senescence	Ethylene biosynthesis	[20]
Strawberry	Infiltration with DETANO Fumigation with NO gas	RT	Delaying postharvest senescence		[21]
Kiwifruit	SNP immersion for 10 min (0.2 mM)	RT	Delaying postharvest senescence	Antioxidant system Cell wall metabolism	[6]
Blueberries	GSNO solution treatment for 30 min (1 mM)	4 °C	Delaying postharvest senescence	Antioxidant system	[22]
Bayberries	NO fumigation (2 h, 20 μL·L^−1^)	1 ± 0.5 °C	Delaying postharvest senescence Alleviating chilling injury	Antioxidant system	[23]
Kiwifruits	NO fumigation (3 h; 10, 20 and 30 μL·L^−1^) NO dip (10 min; 0.5, 1.0 or 2.0 mM)	20 °C	Delaying postharvest senescence Improving postharvest quality	Antioxidant system	[24]
Mango	SNP immersion (5 min, 1.0 mM or 1.5 mM)	8 ± 0.5 °C	Delaying postharvest senescence Alleviating chilling injury	Ethylene biosynthesis	[25]
Mango	NO fumigation (2 h; 5, 10, 20 and 40 μL·L^−1^)	5 ± 1 °C	Delaying postharvest senescence Improving postharvest quality Alleviating chilling injury	Ethylene biosynthesis	[26]
Mango	SNP immersion (5 min, 0.1 mM)	25 °C	Delaying postharvest senescence Improving postharvest quality Inhibiting postharvest diseases		[27]
Banana	SNP immersion (5 min, 0.05 mM)	7 °C	Delaying postharvest senescence		[28]
Banana			Delaying postharvest senescence		[29]
Pistachios	SNP spray (30 s; 15, 30, 45 and 60 μM)	2 ± 1 °C	Delaying postharvest senescence	Antioxidant system	[30]
Jujube	NO fumigation (3 h, 20 μL·L^−1^)	0 ± 1 °C	Delaying postharvest senescence Improving postharvest quality	Antioxidant system	[31]
Litchi	NO dip (5 min; 0.5, 1.0 or 2.0 mM)	32 ± 2 °C	Delaying postharvest senescence Improving postharvest quality		[32]
Cherry	NO dip (5 min; 0.25, 0.5, 1.0 and 1.5 mM)	2 °C	Delaying postharvest senescence	Polyamine metabolism and GABA shunting	[33]
Guava	SNP immersion (5 min; 0.5, 1.0, and 1.5 mM)	20 ± 3 °C	Delaying postharvest senescence Improving postharvest quality	Antioxidant system	[34]
Grape	SNP immersion (5 min, 0.25 and 0.5 Mm)	−0.5 °C	Delaying postharvest senescence Alleviating chilling injury	Antioxidant system	[35]
Pointed gourd	NO dip (10 min, 1.0 and 2.0 mM)	12 °C	Delaying postest senescence Improving postharvest quality	Antioxidant system	[36]
Persimmon	NO dip (30 min, 1.0 and 1.5 mM)	1 °C	Delaying postharvest senescence	Antioxidant system	[37]
Plums	NO fumigation (3 h; 5, 10 and 20 μL·L^−1^)	21 ± 1 °C	Delaying postharvest senescence Improving postharvest quality, alleviating chilling injury		[38]
Plums	Cold storage		Delaying postharvest senescence	Cell wall metabolism	[39]
Peach	NO fumigation (3 h, 10 μL·L^−1^)	4 °C	Delaying postharvest senescence Improving postharvest quality	Sugar metabolism	[40]
Tomato	SNP immersion (30 min, 1 mM)	25 °C	Delaying postharvest senescence Inhibiting postharvest diseases	Ethylene biosynthesis	[41]
Citrus	SNP immersion (2 min, 2%)	20 °C	Delaying postharvest senescence Improving postharvest quality Inhibiting postharvest diseases		[42]
Papaya	NO fumigation (2 h, 20 μL·L^−1^)	20 °C	Delaying postharvest senescence	Cell wall metabolism	[43]
Oranges	NO dip (10 min; 30, 50 and 100 mM)	20 °C	Delaying postharvest senescence Improving postharvest quality Inhibiting postharvest diseases		[44]
Peach	NO fumigation (5 h, 10 μL·L^−1^)	21 ± 1 °C	Improving postharvest quality	Ethylene biosynthesis	[5]
Peach			Improving postharvest quality		[45]
Banana	SNP immersion (5 min, 0.05 mM)	22 ± 1 °C	Improving postharvest quality Alleviating chilling injury	Antioxidant system Energy metabolism	[46]
Apple	NO dip (5 min; 50, 100 and 200 µM)	25 ± 2 °C	Improving postharvest quality Inhibiting postharvest diseases	Antioxidant system	[47]
Kiwifruit	NO dip (10 min; 0.5, 1 and 2 µM)	4 °C	Improving postharvest quality	Antioxidant system	[48]
Winter jujube	NO fumigation (3 h; 10, 20 and 30 μL·L^−1^)	22 °C	Improving postharvest quality		[49]
Peach	NO fumigation (1 h; 5, 10, 20 and 30 μL·L^−1^)	25 °C	Improving postharvest quality		[50]
Oranges	SNP immersion (5 min, 1 mM)	18–23 °C	Improving postharvest quality		[51]
Peach	NO dip (10 min; 0.5, 15 and 100 μM)	20 °C	Inhibiting postharvest diseases		[52]
Tomato	_L_-NNA, NaN 3 (0.5 min, 0.1 mM)	25 ± 1 °C	Inhibiting postharvest diseases		[53]
Pitaya	SNP immersion (8 min, 0.1 mM)	25 °C	Inhibiting postharvest diseases	Cell wall metabolism	[54]
Orange	SNP immersion (5 min, 0.25 or 0.5 mM)	3 °C	Alleviating chilling injury	Antioxidant system	[55]
Banana	NO fumigation (3 h, 60 μL·L^−1^)	7 °C	Alleviating chilling injury	Antioxidant system	[56]
Longkong	SNP immersion (20 min; 10, 20 and 30 mM)	13 °C	Alleviating chilling injury	Antioxidant system	[57]
Hami melon	NO fumigation (3 h, 60 μL·L)	1 + 0.5 °C	Alleviating chilling injury	CBF pathway	[58]
Tomato	NO fumigation (5 h, 200 μL·L^−1^)	20 °C		Ethylene biosynthesis	[59]
Pear	SNP immersion (12 h, 100 μM)	25 °C		Ethylene biosynthesis	[60]
Table grapes	NO fumigation (300 μL·L^−1^)	1 + 0.5 °C		Antioxidant system	[3]
Banana	SNP immersion (5 min, 0.05 mM)	7 °C		Polyamine metabolism and GABA shunting	[61]
Cherry	SNP immersion (20 min; 250, 500 and 1000 μM)	25 °C		Polyamine metabolism and GABA shunting	[33]
Peach	SNP immersion (10 min, 0.50 μM)	4 °C		Cell wall metabolism	[62]
Raspberries				Sugar metabolism	[63]
Apple				Sugar metabolism	[64]
Tomato	SNP immersion (0.5 min, 0.05 and 0.1 mM)	2 ± 1 °C		CBF pathway	[65]
Peach	GSNO immersion (20 min, 60 μM)	23 °C		S-nitrosylation	[66]

Notes: RT, room temperature; SNP, sodium nitroprusside; DETANO, diethylenetriamine-nitric oxide; GSNO, S-nitrosoglutathione.

**Table 2:** Overview of the genes regulated by NO during the postharvest storage of fruits.

Plants	Genes	References
Table grape	*VvSOD*, *VvCAT*, *VvPOD2* and *VvGR*	[3]
Banana	*MaSOD*, *MaCAT*, *MaPOD* and *MaAPX*	[56]
Kiwifruit	*PAL*, *POD* and *CHT*	[6]
Wax apple	*SsC4H*	[19]
Tomato	*LeACO1*, *LeACOH2* and *LeACO4*	[59]
Tomato	*LeACS2*, *LeACS4*, *LeACO1*, *LePG*, *LePhy1* and *LePME*	[41]
Pear	*PcCel*, *PcPG*, *PcACS*, *PcACO* and *PcNOS*	[60]
Tomato	*LeCBF1*	[65]
Hami melon	*CmCBF1* and *CmCBF3*	[58]
Apple	*MdSPS*, *MdSS*, *MdNI*, *HK* and *FK*	[64]
Peach	*PpaSPS1/2 PpaAI1*	[40]
Peach	*GSNOR PR1*, *NPR1* and *TGA1*	[66]

## Data Availability

Not applicable.
